# Comparison of binder compositions in Pompeian wall painting styles from *Insula Occidentalis*

**DOI:** 10.1186/s13065-014-0065-0

**Published:** 2014-11-20

**Authors:** Monica Gelzo, Mario Grimaldi, Alessandro Vergara, Valeria Severino, Angela Chambery, Antonio Dello Russo, Ciro Piccioli, Gaetano Corso, Paolo Arcari

**Affiliations:** Dipartimento di Medicina Molecolare e Biotecnologie Mediche, Università di Napoli Federico II, Via Pansini 5, I-80131 Naples, Italy; Centro Internazionale per gli Studi Pompeiani, Università Suor Orsola Benincasa, Via Suor Orsola 10, I-80135 Naples, Italy; Dipartimento di Scienze Chimiche, Università di Napoli Federico II, Via Cintia 21, I-80126 Naples, Italy; Distretto ad Alta Tecnologia dei Beni Culturali (DATABENC) Scarl, Naples, Italy; Dipartimento di Scienze e Tecnologie Ambientali, Biologiche e Farmaceutiche, Seconda Università di Napoli, Via Vivaldi 43, I-81100 Caserta, Italy; AIES Beni culturali, I-80055 Portici, Napoli, Italy; Dipartimento di Medicina Clinica e Sperimentale, Università di Foggia, Via Pinto 1, I-71122 Foggia, Italy; CEINGE, Biotecnologie Avanzate Scarl, Via Comunale Margherita, 482 I-80145 Naples, Italy

**Keywords:** Pompeian wall painting, Binder analysis, Spectroscopy, Mass spectrometry, Cultural heritage

## Abstract

**Background:**

Although the pigment composition of Pompeian wall paintings has been the object of several studies, a comprehensive characterization of paint binder components is still lacking. This work aimed investigated at a molecular level the binder composition differences among wall paintings belonging to different periods of Pompeii’s history. Analytical investigations were performed on representative samples of the first, second, third, and fourth painting styles excavated from the house of *Marcus Fabius Rufus* (*Insula Occidentalis*). The application of sensitive experimental methodologies was complemented by historical knowledge to gain insight in painting techniques and materials used by Pompeian artists.

**Results:**

Fourier transform infrared spectroscopy and Raman spectroscopy were used to investigate the organic components and pigments present in powders obtained from samples of the four painting styles. No proteinaceous components were detected in the samples with liquid chromatography-electrospray ionization-hybrid quadrupole/time-of-flight mass spectrometry. Liquid chromatography, gas chromatography with flame-ionization detection, and gas chromatography–mass spectrometry of polar and non-polar components extracted from powders were used to evaluate and compare the free amino acids, sugars, and fatty acids profiles.

**Conclusions:**

Pigments and natural products (lipids, gums and wheat flours) were the main components of all samples. This supports the hypothesis that artists likely used water tempera for Pompeian wall paintings.

**Graphical Abstract:**
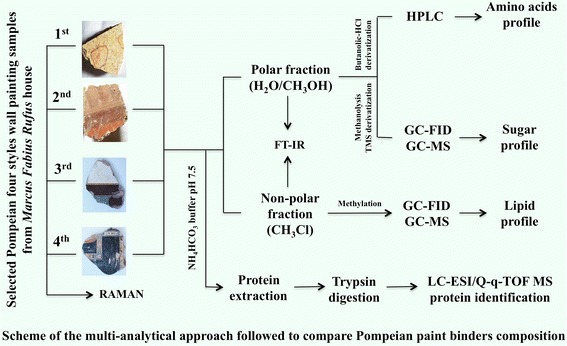
Scheme of the multi-analytical approach followed to compare Pompeian paint binders composition.Scheme of the multi-analytical approach followed to compare Pompeian paint binders composition.

**Electronic supplementary material:**

The online version of this article (doi:10.1186/s13065-014-0065-0) contains supplementary material, which is available to authorized users.

## Background

The house of *Marcus Fabius Rufus*, *Insula Occidentalis,* is one of the most remarkable examples in the architectural landscape of Pompeii. On four levels down to the sea, the house can be considered as a typical villa in the city. The garden is located on the western side of the house, near to the city walls made of ashlar in Sarno limestone. Most of the house, except for the central circular exedra, was destroyed in the 79 A.D. eruption. This area was initially superficially explored in the Bourbon Restoration period, and some decorations were removed and placed in the National Archaeological Museum of Naples [1]. Following a survey of the upper floor of the house by the La Vega brothers (1787–1809), later investigations by Amedeo Maiuri [2] sought to restore the house. At the end of the 1970s, additional excavations uncovered the outer garden on the west slope and further restorations were carried out [3]. Since 2004, *Marcus Fabius Rufus* house and associated areas have been subject to further investigations [1,4,5] and excavations in the garden area [6-8]. Wall paintings found within the house and in neighboring buildings have been studied [9]. These studies have aimed to reconstruct the urbanization phases of this part of ancient Pompeii, which represents an ideal model to unravel the occupation phases of the *Insula Occidentalis*.

In this context, the study of aged paintings is a very challenging task that requires the knowledge of both analytical techniques and historical and conservation techniques [10,11].

In this work, we investigated and compared at a molecular level the binder compositions of wall paintings belonging to different periods of Pompeii’s history. This was performed to gain novel insights with respect to previous recent publications on archaeometric studies of Roman paintings [12-21]. Pompeian wall painting samples were collected during excavations carried out in the garden area outside *Marcus Fabius Rufus* house and in the terrace above the *Villa Imperiale*. These samples were classified as belonging to the first, second, third, and fourth Pompeian decoration styles by the archaeologist M. Grimaldi [1]. The Pompeian *Villa* was almost completely restored after the earthquake of 62 A.D. Successive renovations caused the partial destruction of earlier room decorations of the fourth, third, and second styles, and the discarded material was used to raise the floor level of the garden. Recent garden excavations (2007–2008) were extended to the levels (4.5 m) of the late Republican period, characterized by the presence of a large painted plaster of the first style [7,8]. Therefore, all excavated wall painting specimens should have been preserved from potential deterioration caused by the subsequent eruption of Vesuvius in 79 A.D. In addition, based on our results, these samples did not contain waxes, components often used in the past for maintenance works carried out to restore the original painting colors [22,23]. Taking into account these considerations, the selected decorated fragments were considered suitable for a comparative analysis of the binder ingredients used by the artists over a wide period spanning from 200 B.C. up to 79 A.D. Complementary analytical techniques, including Raman and Fourier-transform infrared (FT-IR) spectroscopy, liquid chromatography-electrospray ionization-hybrid quadrupole/time-of-flight mass spectrometry, liquid chromatography, gas chromatography (GC) with flame-ionization detection, and gas chromatography–mass spectrometry (GC-MS) were used to analyze and compare the pigment and binder (polar and non-polar) compositions of the representative mural painting samples.

### Experimental

#### Materials and reagents

Analytical high-performance liquid chromatography (HPLC) solvents, including acetonitrile, formic acid, methanol, ethanol, dichloromethane, chloroform, and pyridine were obtained from JT Baker (Deventer, Netherlands). Sodium dihydrogen phosphate monohydrate, and hydrochloric acid (HCl) 37% were purchased from Carlo Erba (Cornaredo, Italy). Potassium hydroxide and ammonium bicarbonate were purchased from Merck (Merck KGaA, Darmstadt, Germany). Purified water was prepared using a Milli-Q system (Millipore Corporation, Billerica, MA). All other reagents were of analytical grade. Amino acid calibration standards in 0.1 mol/L HCl, borate buffer 0.4 mol/L in water (pH 10.2), and 10 mg/mL *o*-phthalaldehyde-3-mercaptopropionic acid (OPA-3-MPA) reagent in borate buffer (0.4 mol/L) were obtained from Agilent Technologies GmbH & Co.KG (Waldbronn, Germany). d-Ribose (Carlo Erba Reagents) was dissolved in distilled water to a final concentration of 1 mg/mL. Bis(trimethylsilyl)trifluoroacetamide and dithiothreitol (DTT) were purchased from Sigma Aldrich (St. Louis, MO). Boron trifluoride/methanol (10%, w/w) was purchased from Supelco (Bellefonte, PA). Acetyl chloride was from Carlo Erba Reagents.

#### Wall painting samples and sample preparation

Specimens were chosen from a collection of wall painting samples stored in the lower part of the excavated *Marcus Fabius Rufus* house in Pompeii (*Insula Occidentalis*). The specimens (about 5x3x2 cm) were carefully handled to prevent contamination. The specimens were of the first (yellow, Y; from 200 B.C. to 90/80 B.C.), second (light red, LR; from 90/80 B.C. to the end of the first century), third (dark red, DR; from the end of the first century B.C. to about half of the first century A.D.), and fourth (black, B; from 35/45 A.D. to the last decades of the first century A.D.) styles. For chemical analyses, powder samples (50 mg) were scraped with a scalpel from the wall painting surface. A combined extraction of polar and nonpolar compounds was carried out according to the method of the Standard Metabolic Reporting Structures working group [24]. Briefly, d-ribose (100 μg) was added to each powder as an internal standard for sugar analysis. Methanol (8 mL/g of powder) and water (1.70 mL/g of powder) were added and the samples were vortex mixed for 3 min. Then, chloroform (4 mL/g of powder) was added, and the samples were incubated on ice for 10 min. Finally, chloroform (4 mL/g of powder) and water (4 mL/g of powder) were added to the samples and, after vortex mixing for 3 min, the samples were centrifuged at 12,000 × *g* for 15 min at 4°C. The upper layer (polar phase; about 0.7 mL) and the lower layer (lipophilic phase; about 0.4 mL) were each dried under a stream of nitrogen (N_2_) and then dissolved in water (50 μL) or chloroform/methanol (2:1, v/v; 50 μL), respectively [25,26].

#### FT-IR and Raman spectroscopy

For FT-IR, an aliquot (5 μL) of each polar or non-polar fraction was dropped onto a 3 mm zinc selenide window, dried under a white lamp (60 W) and analyzed with a Nicolet 5700 equipped with a ContinuμM™ infrared microscope (Thermo Fisher Scientific, Waltham, MA). For each sample, spectra (200 acquisitions) were collected in transmission mode, with a sensitivity of eight, and the microscope focusing windows set at 100 × 100 μm. Spectra were analyzed using Omnic software (Thermo Fisher Scientific). Peaks were assigned by comparison to spectral databases [27].

Raman spectra were recorded using a confocal Raman microscope (NRS-3100, Jasco Applied Sciences, Halifax, Canada). The 647 nm line of a water-cooled Kr^+^ laser (Innova 302, Coherent, Santa Clara, CA) at 400 mW was injected into an integrated Olympus microscope and focused to a spot size of approximately 2 μm (100× or 20× objective). The laser power at the sample ranged from 1 to 10 mW depending on the sample photosensitivity. The spectral resolution was 4 cm^−1^. Raman spectra were recorded at three separate spots on each paint powder to evaluate the heterogeneity. A holographic notch filter was used to reject the excitation laser line. Raman scattering was collected by a Peltier-cooled charge-coupled device photon detector (DU401BVI, Andor Technology, Belfast, Northern Ireland). For most of the spectra, a complete data set was collected in 100 s.

#### Amino acid analysis

The samples were subjected to a precolumn derivatization in the needle of the autosampler with OPA-3-MPA, and injected in the HPLC system as previously reported [28]. Briefly, each sample (25 μL) was transferred into a conical vial insert for precolumn derivatization, and the amino acid concentration was determined using the calibration curve. Amino acids were identified and quantified by comparison of their retention time and absorption ratio with those of authentic compounds in the calibration solution. The analyses were performed using an Agilent Technologies 1200 Series LC System (Agilent, Santa Clara, CA) equipped with a binary pump delivery system, an autosampler to automate the precolumn derivatization and injection procedure, a heated column compartment, and a programmable fluorescence detector. All of the equipment was controlled by Agilent ChemStation software. An Agilent Zorbax Eclipse XDB-C18 analytical column (5 μm, 4.6 × 150 mm), was used in parallel to an Agilent Eclipse XDB-C18 analytical Guard column (5 μm, 4.6 × 12.5 mm) for chromatographic separations. The HPLC retention times of all amino acids are reported in Additional file 1: Table S4.

#### Mass spectrometry analysis

*Sugar and lipid analysis -* Sugar analysis was performed as previously reported [25,26]. Briefly, 35 μL of the polar extract solution was dried under a N_2_ stream. The residue was resuspended in 0.5 mL of a methanolic HCl solution prepared by adding acetyl chloride (0.4 mL) to 15 mL of methanol. Methanolysis was conducted at 80°C for 24 h. Thereafter, the solvent was removed using a N_2_ stream, the residue was derivatized using a mixture of pyridine and *N*,*O*-bis(trimethylsilyl)trifluoroacetamide (0.2 mL, 3:7). The solution was heated at 80°C for 30 min. The derivatized sample was dried under a N_2_ stream and the residue was dissolved in 50 μL of methylene chloride (CH_2_Cl_2_). Aliquots (1 μL) of the samples were analyzed by gas chromatography with flame-ionization detection (GC-FID) and GC-MS.

For lipid analysis, the chloroform extract solution (45 μL) was dried under a N_2_ stream and re-suspended in 1 mL of BF_3_/methanol (10%, w/w). Methylation was performed at 60°C for 10 min. Then, the sample was mixed with 1 mL of distilled water and the lipids were extracted two times with 1 mL of hexane. The upper layers (lipophilic phases) were pooled and 10 μg of methyl heptadecanoate was added as an external standard. The samples were dried under a gentle N_2_ stream and the residue was dissolved in 50 μL of CH_2_Cl_2_. Aliquots (1 μL) of the samples were analyzed by GC-FID and GC-MS.

Both sugars and lipids were analyzed by GC-FID (HP-5890, Agilent) and GC-MS (GC 8000/MD800, Fisons Instruments) controlled by a workstation equipped with MassLab 3.4 software [25,26].

*Protein analysis -* For protein analysis, the four painting powder samples (about 8–10 mg) were suspended in 100 μL of 50 mmol/L ammonium bicarbonate, sonicated, and incubated in 5.8 mmol/L dithiothreitol for 5 min at 95°C for reduction of disulfide bridges. The total protein tryptic digest, obtained as previously described [29], was dried, re-suspended in 20 μL of 0.1% formic acid in water and analyzed by LC-MS using a CapLC system directly connected to a modular hybrid quadrupole-orthogonal time-of-flight mass spectrometer (Micro Q-TOF; Waters, Milford, MA) equipped with a Z-spray source [29].

The identification of proteinaceous material in the four samples was performed by principal component analysis (PCA), using the amino acidic percentage content and the contents of 15 representative proteins from milk, wheat flour (gluten, gliadin, gutenin, albumin), egg (lysozyme, conalbumin, ovomucoid, avidin, phosvitin, vitellin) and glue (crocodile, python, toad, chicken) [30-33]. Rabbit and horse proteins were not included in this analysis. XLSTAT statistical analysis software (Addinsoft, New York, NY) was used.

#### Data analysis

The signal-to-noise (S/N) ratio approach was used to estimate the limit of detection (LOD) and the limit of quantitation (LOQ) for amino acids, lipids and sugars on three blank replicates. The LOD and LOQ were calculated based on the chromatographic responses of the analytes as the average plus three and six times the standard deviation, respectively. The LOD and LOQ values for the analyzed compounds are reported in Additional file 1: Table S8.

## Results and discussion

**Figure 1:**
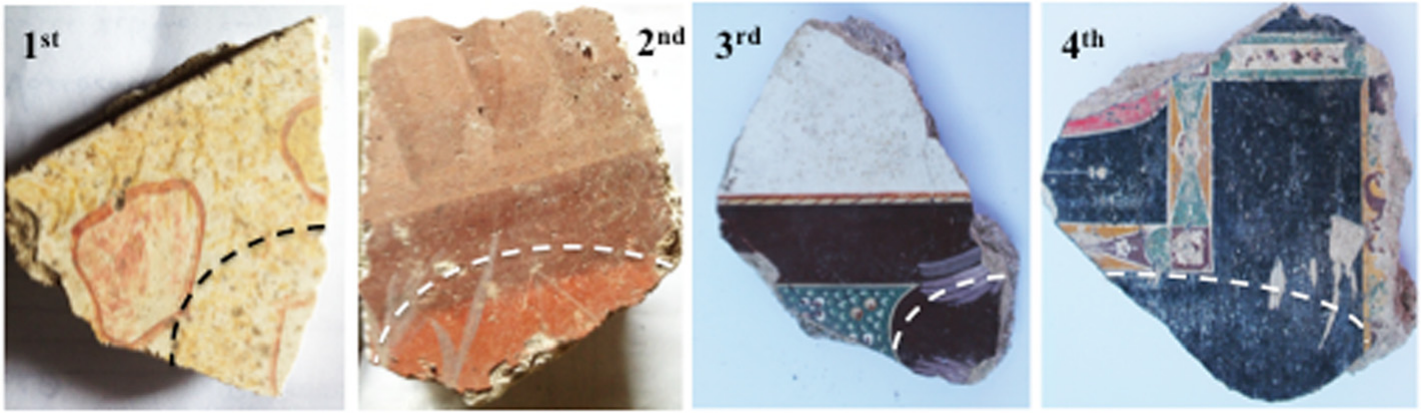
**Pompeii wall painting styles.** Representative fragments belonging to the first, second, third and fourth decoration styles were collected from *Marcus Fabius Rufus’s* house. The sampling area for chemical analysis is indicated by a dashed line.

**Figure 2:**
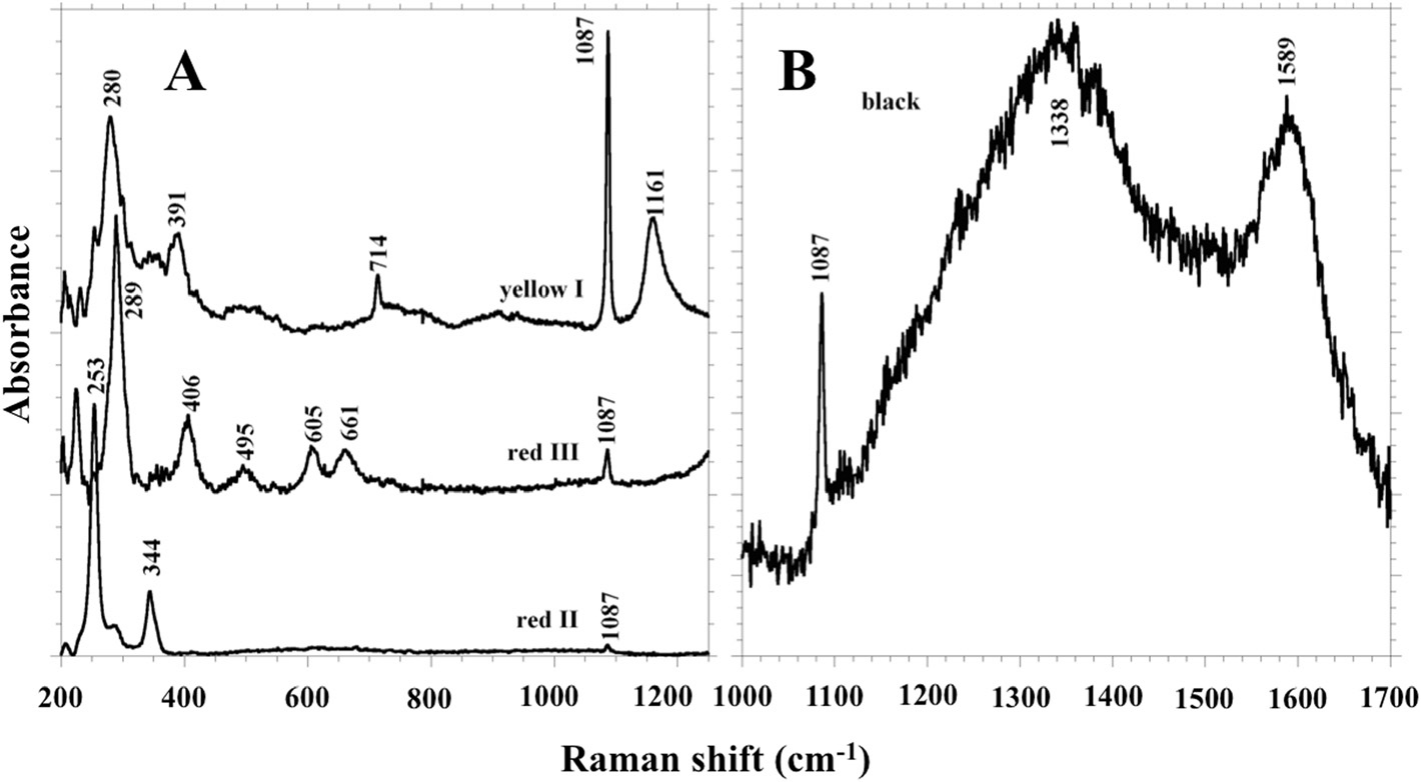
**Raman spectra of Pompeii samples. A**. Yellow I = first style; Red II = second style; and Red III = third style. **B**. Black = fourth style.

Representative pictures of the selected wall paintings specimens excavated from the *Insula Occidentalis* in Pompeii are shown in Figure 1. Raman spectra recorded from sample powders (Figure 2) showed signals from plaster and pigment in all samples. The spatial heterogeneity analysis showed only a slight variation in the relative intensity of these two major components. Furthermore, in all samples, peaks were present at 1087 and 714 cm^−1^, which were assigned to the presence of calcite (CaCO_3_) [34,35]. No Raman signals were observed for other carbonates (e.g. 1098 cm^−1^ of dolomite). No gypsum signal was observed around 1007 cm^−1^, which suggests a good state of conservation [34]. The first style sample (Y) showed Raman features suggesting a carotenoid structure. In particular, the frequencies (1161 and 1521 cm^−1^, medium frequency region not shown) and the relative intensities of these two bands (almost 1:1) were typical of all trans β-carotene [36,37], previously described in Roman paintings [38]. The second style sample (LR) showed very strong signals at 253 and 344 cm^−1^, which were assigned to the cinnabar (HgS) [39,40] contained in red vermillion, as also reported for other Roman wall paintings [40]. The third style sample (DR) was characterized by signals at 289, 406, 495, 605 and 661 cm^−1^, indicating a mixture of iron oxides, which are typical of red ochre pigment [40,41]. The fourth style sample (B) (Figure 2B), showed two weak and broad bands around 1338 and 1589 cm^−1^ that could be assigned to C-C stretching modes of amorphous carbon [41,42]. The absence of a band at 960 cm^−1^, could be attributed to symmetric stretching modes of the phosphate fragments in calcium phosphate. This suggests that the carbon/charcoal was likely extracted from vegetable sources, although animal material cannot be ruled out. Additional file 1: Table S1 shows all the Raman bands observed in the spectra and the peak assignments.

The presence of proteins was evaluated by shotgun LC-MS/MS analysis directly on powder samples after tryptic digestion [29]. No proteinaceous material was detected by liquid chromatography-electrospray ionization-hybrid quadrupole/time-of-flight mass spectrometry, even though this technique has high sensitivity. The potential formation of protein complexes with metal ions or other materials may influence and prevent extraction of suitable amounts of protein for the LC-MS/MS analysis. However, our results confirmed previous findings on Pompeian wall paintings [25] and suggested that the painting mixture was probably made of pigments dissolved in a liquid medium containing organic components of vegetable and plant origin. Casoli et al., 2012 [43] also reported the absence of proteinaceous material in fresco paint samples taken from *Insula del Centenario*. By contrast, proteinaceous materials were present in restored paint samples but, in this study, the amino acid content was determined by GC-MS after acid hydrolysis of the samples [43].

In the present work, the polar and non-polar fractions were extracted from the painting samples with water/methanol and chloroform, respectively. These fractions were analyzed to detect the presence of free amino acids, sugars and lipids.

**Figure 3:**
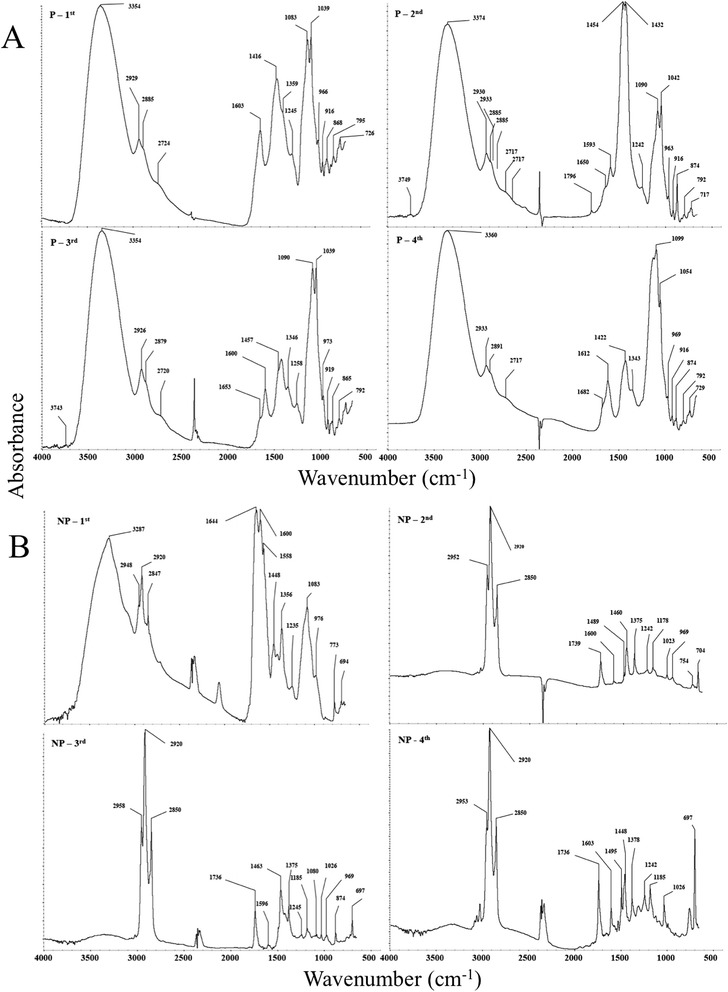
**FT-IR spectra of Pompeii samples. A**. FT-IR spectra of the polar fractions (P). **B**. FT-IR spectra of the non-polar fractions (NP).

The FT-IR analysis of the polar fraction (water/methanol) showed several major bands attributable to organic compounds (Figure 3A) [44]. The main peaks corresponded to the stretching of the hydroxyl group (−OH) (weak, around 3740 cm^−1^) [27], N-H (region 3500–3300 cm^−1^) and C-H (region 2950–2880 cm^−1^). The C-H stretch indicated the presence of carbon chains. Amide I and amide II bands (1648 cm^−1^ and 1595 cm^−1^, respectively) were weak or not detected, which indicates the absence of proteins. The intense absorption band around 1045 cm^−1^ suggested the presence of a carbohydrate side group (COH) [45]. As expected, and in agreement with the Raman analysis, characteristic bands of calcite (1790, 1456, 1090, 874, 717 cm^−1^) were observed [44].

The FT-IR spectra of non-polar fractions of the first style sample (Figure 3B) showed a different profile compared to those of the other three styles, with the presence of bands similar to those of terpenoid derivatives [36,46]. This is consistent with the Raman result. Typical spectra of natural resins show a broad band in the 3287 cm^−1^ region that arises from stretching of the -OH group, a strong band at 1644 cm^−1^ for the absorption of carbonyl groups (C = O), and a weak band at 1235 cm^−1^ for the carbon-oxygen bond (C-O). The spectra of the second, third, and fourth styles showed profiles typical of oils. These spectra were characterized by bands in 2950–2847 cm^−1^ (functional groups region), 1736–1558 cm^−1^ (double bond stretching), and 1448–1356 cm^−1^ (double bond deformation) regions because of C-H bending [47]. All the major FT-IR bands observed and the peak assignments are shown in Additional file 1: Table S2 and Table S3.

**Figure 4:**
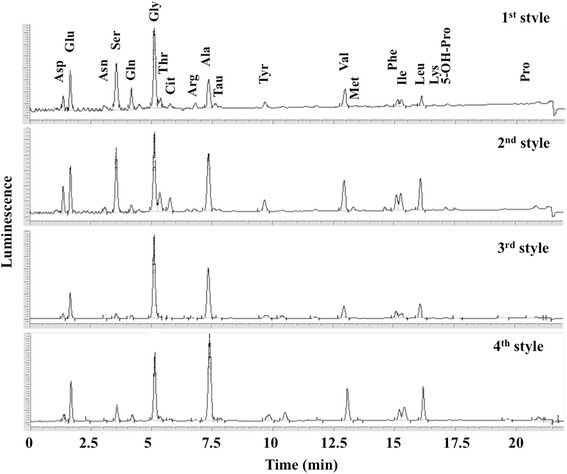
**Free amino acid HPLC profiles of Pompeii samples.** Polar fractions were analyzed in duplicate by a spectrofluorimetric method following derivatization of amino acids with OPA-3-MPA.

**Figure 5:**
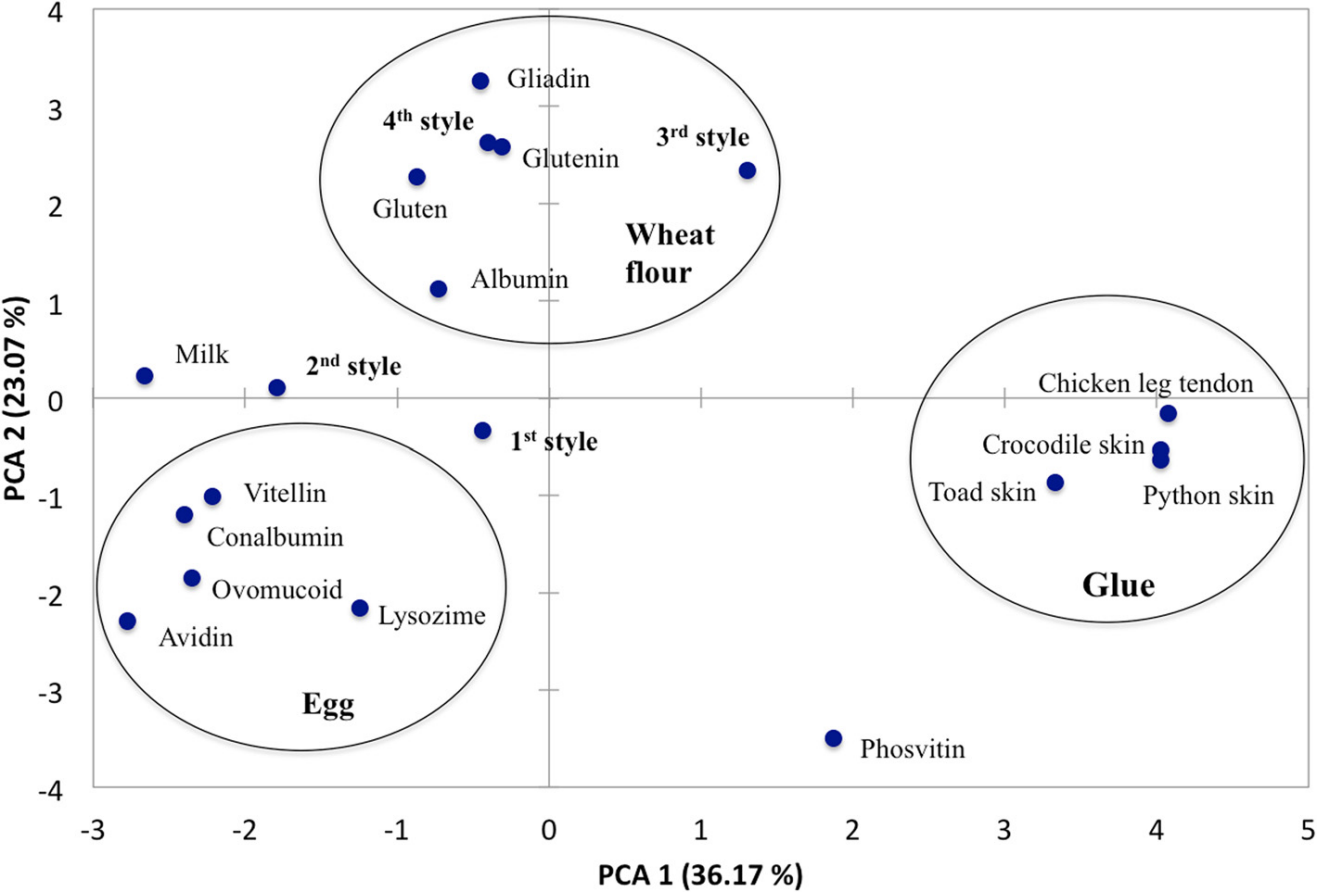
**PCA score plot obtained based on the amino acid contents for the four Pompeian samples and 15 reference proteins.** The relative percentages of amino acids in the Pompeii samples and of protein from wheat flour, egg, animal glue and milk are reported in Additional file 1: Tables S4 and S5.

The free amino acid elution profile for the polar fractions is reported in Figure 4. The concentrations found in the four samples, expressed as milligrams per kilogram of powder and as percentages, are reported in Additional file 1: Table S4. Among the 20 amino acids detected, the most abundant in all samples were Glu, Gln, Val, Pro, Ala, Ser and Gly. Similar profiles were reported for cereals [25]. The amino acid content was markedly lower in the older samples of the first and second styles than the samples of the third and fourth styles (Additional file 1: Figure S1). To verify whether the observed free amino acid profiles were consistent with the presence of proteinaceous materials, such as wheat flour, egg, animal glues and milk, in the original painting (Additional file 1: Table S5), a principal component analysis (PCA) comparison was performed between the amino acid composition determined in the four Pompeian samples and the amino acid composition of reference proteins [43]. The first two components (Figure 5, PCA1 and PCA2) accounted for 59.2% of the variance in the data, and grouped the data into three clusters. The upper-left cluster (Figure 5) showed that the amino acid compositions of samples from to third and fourth styles overlapped with that of wheat flour protein (gliadin, glutenin and gluten), while the amino acid compositions of the first and second styles did not overlap with any proteins (wheat, egg or animal glue) (Figure 5). The third and fourth style samples could be discriminated using alanine, glycine, leucine/isoleucine, proline, glutamic acid, phenylalanine, and tyrosine. To confirm this finding, the percentages of the amino acids in the third and fourth style samples were submitted to Swiss-Prot and/or TrEMBL AACompIdent to identify proteins from the amino acid compositions [48]. Although none of the clustered natural proteins used for the comparison was identified by this bioinformatic approach, it is possible that our analysis was affected by the presence of several metal ions, including mercury (Hg^2+^) in the second style sample and iron (Fe^3+^) in the third style sample, and calcium (Ca^2+^) from calcite in all samples. Amino acids have different affinities for various ions, and the identification of proteinaceous materials in the samples using the amino acid profiles may have been hampered by the strong affinity of amino acids for these metal ions. During aging of the paintings, metal ions and amino acids could form highly insoluble complexes.

**Figure 6:**
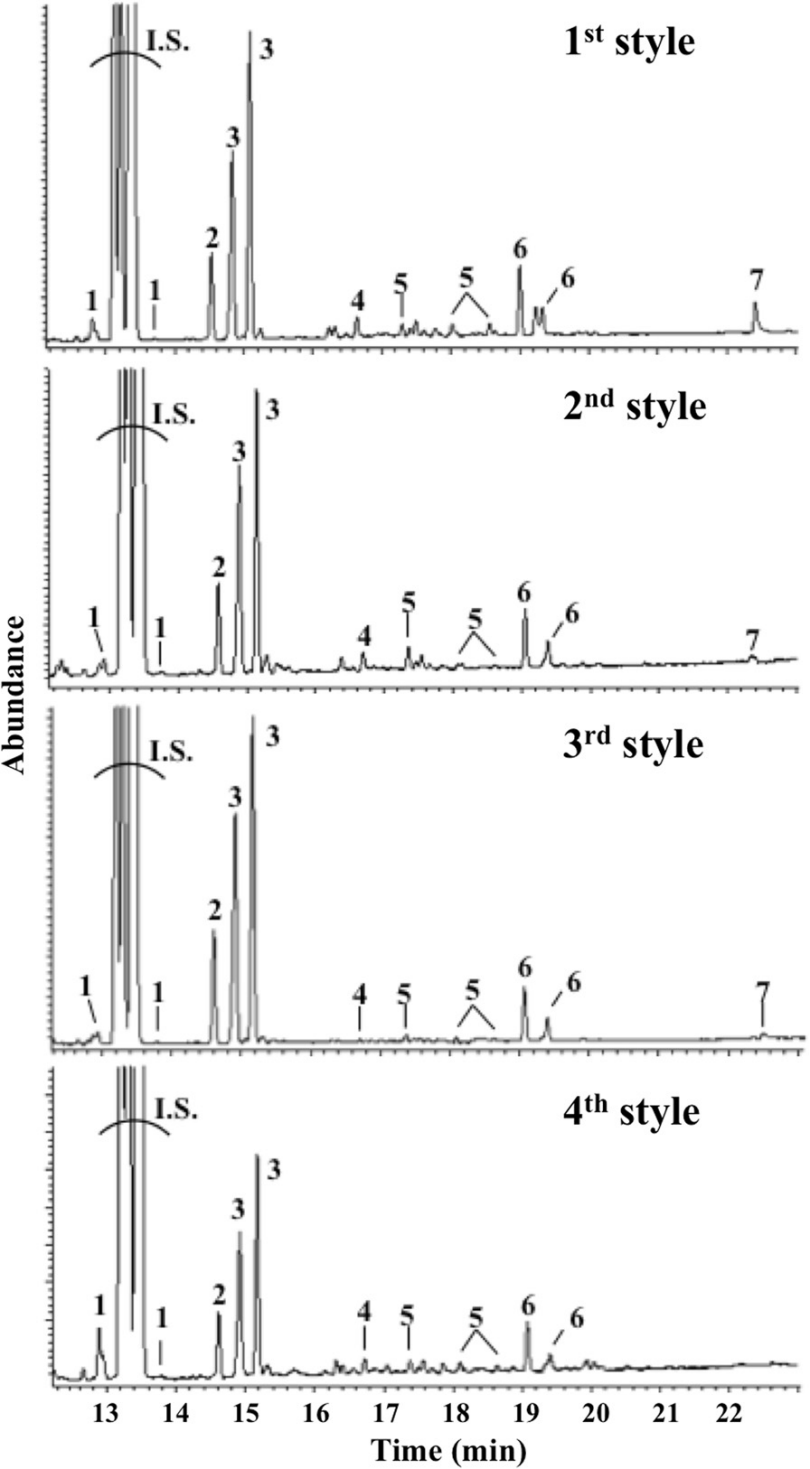
**GC-FID analyses of trimethylsilylated sugars from polar extracts of the first, second, third, and fourth style samples.** Peaks: 1, arabinose; 2, fucose; 3, xylose; 4, galacturonic acid; 5, galactose; 6, glucose; 7, myo-inositol. I.S. = internal standard (d-ribose).

Analyses of sugars in the polar fractions are reported in Figure 6 and Additional file 1: Table S6. The GC profile showed several peaks, which were identified as arabinose, fucose, xylose, galactose, glucose, galacturonic acid, and myo-inositol. Among these sugars, xylose was the most abundant in all samples (average 64% of total sugar content). The sugar contents (in mg/kg) are shown in Additional file 1: Figure S2. The myo-inositol content decreased from the oldest samples to the younger samples as follows: first style, about 5%; second style, about 1%; third style, about 0.3%; and fourth style, <0.1% (Additional file 1: Figure S3). This suggests a correlation between myo-inositol and the sample age and state of conservation. As previously suggested by Bonaduce et al., myo-inositol can be present in samples because of bacterial or fungal growth on mural paintings [49]. In addition, it cannot be ruled out that contamination by these agents has also altered the protein/amino acid content of the samples.

The ranges of the ratios of [fucose/(arabinose + xylose)] and [galactose/(arabinose + xylose)] were 0.15–0.18 and 0.02–0.07, respectively (Additional file 1: Table S6). Although our results are in agreement with previous reports on the sugar contents in mural paintings [49], the high content of xylose (xylose/arabinose ratio >1) suggested tragacanth and fruit tree gums were not present because these gums have a ratio of <1. The absence of rhamnose in our samples indicates the absence of tree gums, at least gum arabic [50], whereas the presence of fucose strongly suggests the presence of tragacanth-type gums [51]. Based on the classification of Reido et al. [52], our findings support the hypothesis that tragacanth gums were used in the paint mixtures.

**Figure 7:**
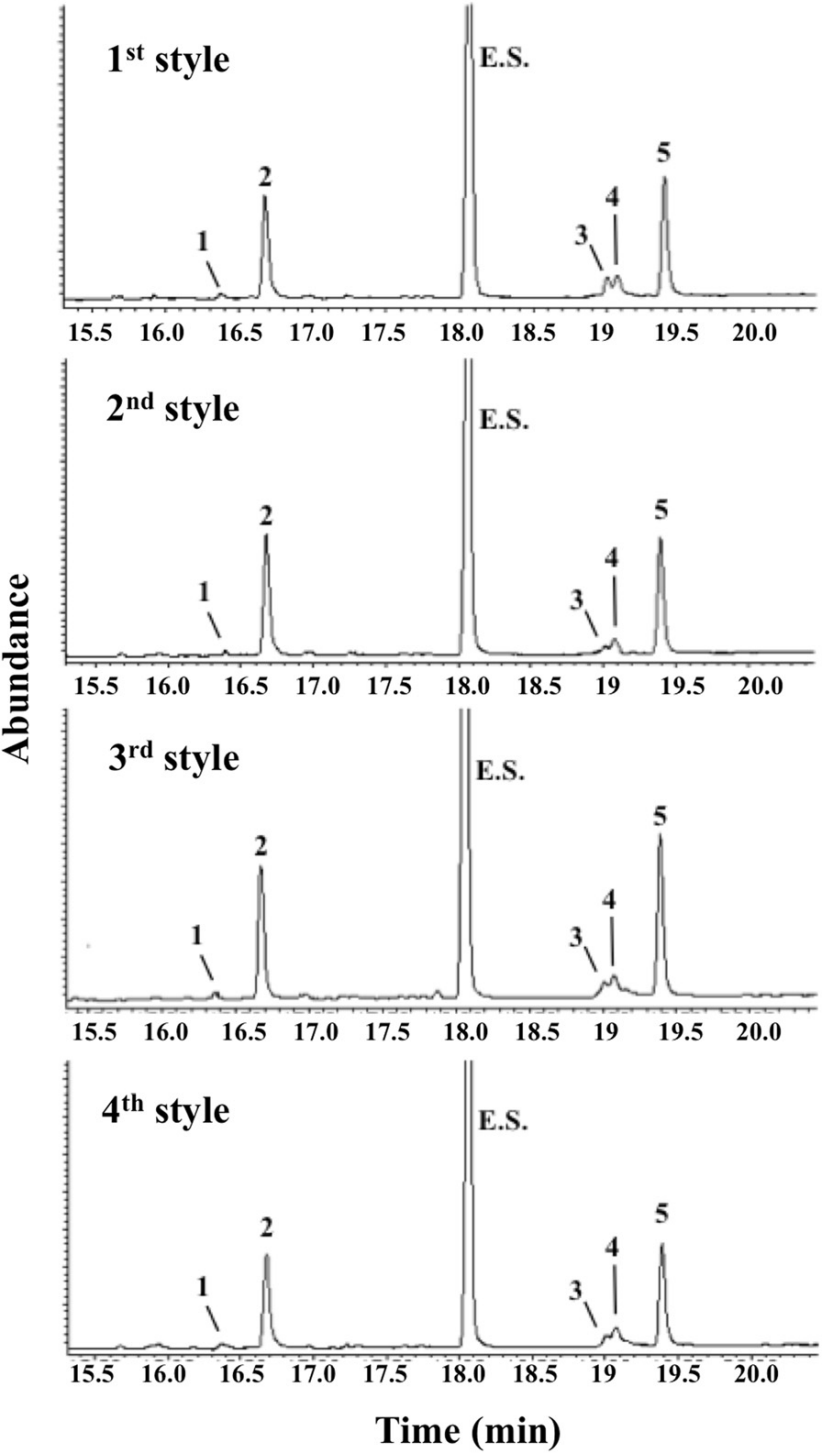
**GC-FID analyses of methylated fatty acids from non-polar extracts of the first, second, third, and fourth style samples.** Peaks: 1, C16:1; 2, C16:0; 3, C18:2; 4, C18:1; 5, C18:0. E.S = external standard (methyl heptadecanoate).

The analysis of non-polar fractions allowed the identification of five fatty acids (Figure 7 and Additional file 1: Table S7), which were identified as C16:1, C16:0, C18:2, C18:1, and C18:0. Other minor peaks, attributed to contaminants (e.g. phthalate) or unidentified compounds, were not considered for calculations. Terpenoids, which were identified by Raman and FT-IR spectroscopy in the non-polar fraction, were not detected by GC. The total content of fatty acids is reported in Additional file 1: Figure S4. The lipid contents were different among the four samples, with C16:0, and C18:0 being the most abundant lipids at 42 and 44%, respectively. The lipids C16:1, C18:2, and C18:1 accounted for 1.6%, 4.9%, and 7.7%, respectively, of the total lipid content. These findings suggest that a mixture of oils of different origins was likely used for preparing the paint. These results are similar to those reported by Duran et al. [23]. However, the present data do not suggest waxes were used in the binder as no even or odd-numbered linear hydrocarbons above C20:0 were detected before or after methylation [53].

## Conclusions

Pompeian wall paintings from four different historical periods were found to contain similar organic binder ingredients with quantitative differences in their chemical compositions. Compared to the other styles, the first style contained more lipids and sugars, while the third and fourth styles contained more amino acids. These differences could be ascribed to paint deterioration because of aging and environmental degradation. The composition could also have been influenced by specific choices of the artists in preparing their paints, such as the technique and binders used to mix colors to improve handling, appearance and color brightness. Because these results are based on the analysis of only four representative samples, further investigations are needed to confirm the observed differences. Especially considering the challenge of identification of proteinaceous material in paint samples because of the strong affinity of amino acids for metal ions in pigments and lime. The use of animal or vegetable proteins in the naive painting mixtures prepared by artists requires further study.

It is interesting to note that the abundance of components detected in the third style sample compared to the second and fourth styles might be linked to the historical context. Indeed, under Emperor Augustus (27 B.C.–14 A.D.) in the second half of the first century B.C., there was an impulse to innovate architecture, sculpture, and painting. Future investigations on different archaeological samples will allow new perspectives in art and archaeological fields and highlight changes in painting techniques. These results can also be used to study human habits from historic and economic points of view [54].

## Additional file

Additional file 1:
**Raman and FT-IR frequencies.** Free amino acid analysis. Mass spectrometry analysis of sugars and fatty acids. LOD and LOQ values for the analyzed compounds.

## Abbreviations

BSTFA: N,O-bis(trimethylsilyl)trifluoroacetamide
CCD: Charge-coupled device
DTT: Dithiothreitol
ESI: Electrospray
FID: Flame-ionization detector
FT-IR: Fourier transform infrared spectroscopy
GC: Gas chromatography
HPLC: High pressure liquid chromatography
LC-ESI/Q-q-TOF MS: Liquid chromatography-electrospray ionization-hybrid quadrupole/time-of-flight mass spectrometry
LOD: Limit of detection
LOQ: Limit of quantitation
MS: Mass spectrometry
OPA-3-MPA: o-phthaladehyde; 3-mercaptopropionic acid
TMS: Trimethylsilyl
TPCK: L-1-Tosylamide-2-phenylethyl chloromethyl ketone
