# Supplementary material for: Comparison of binder compositions in Pompeian wall painting styles from Insula Occidentalis
Source: Chem Cent J. 2014 Nov 20;8:65. doi: 10.1186/s13065-014-0065-0 (PMC4240868; doi:10.1186/s13065-014-0065-0)
Supplement: Additional file 1: — Raman and FT-IR frequencies. Free amino acid analysis. Mass spectrometry analysis of sugars and fatty acids. LOD and LOQ values for the analyzed compounds. [file 13065_2014_65_MOESM1_ESM.doc]

**Additional file 1**

**Comparison of binder compositions in Pompeian wall painting styles from *Insula Occidentalis***

Monica Gelzo^1^, Mario Grimaldi^2^, Alessandro Vergara^3,4^, Valeria Severino^5^, Angela Chambery^5^, Antonio Dello Russo^1^, Ciro Piccioli^6^, Gaetano Corso^7*^, Paolo Arcari^1,8*^

^1^Dipartimento di Medicina Molecolare e Biotecnologie Mediche, Università di Napoli Federico II, Via Pansini 5, I-80131 Napoli, Italy

^2^Centro Internazionale per gli Studi Pompeiani, Università Suor Orsola Benincasa, Via Suor Orsola 10, I-80135 Napoli, Italy

^3^Dipartimento di Scienze Chimiche, Università di Napoli Federico II, Via Cintia 21, I-80100 Napoli, Italy

^4^Distretto ad Alta Tecnologia dei Beni Culturali (DATABENC) Scarl, Napoli, Italy

^5^Dipartimento di Scienze e Tecnologie Ambientali, Biologiche e Farmaceutiche, Seconda Università di Napoli, Via Vivaldi 43, I-81100 Caserta, Italy

^6^AIES Beni culturali, I-80055 Portici, Napoli, Italy

^7^Dipartimento di Medicina Clinica e Sperimentale, Università di Foggia, Via Pinto 1, I-71122 Foggia, Italy

^8^CEINGE, Biotecnologie Avanzate s.c.a.r.l, Via Comunale Margherita, 482 I-80145 Napoli, Italy

* Correspondence: [arcari@unina.it](mailto:arcari@unina.it), g.corso@unifg.it

**Raman and FT-IR frequencies.**

**Table S1. Raman frequencies observed in the paint powder from** Pompeii’s samples and peak assignments

| Frequency cm^-1^ | | | | Predicted peak |
| --- | --- | --- | --- | --- |
| 1^st^- Yellow | 2^nd^ - Red | 3^d^ – Red | 4^th^- Black |  |
| ---- | ---- | ---- | 1589 | Large carbon-based peak^a,b^ |
| 1521 | ---- | ---- | ---- | *ν* C=C^c,d^ |
| ---- | ---- | ---- | 1338 | Large carbon-based peak^a,b^ |
| 1161 | ---- | ---- | ---- | *ν* C-C^c,d^ |
| 1087 | 1087 | 1087 | 1087 | Calcite^a,e,f^ |
| 714 | ---- | ---- | ---- | Calcite^a,e,f^ |
| ---- | ---- | 661 | ---- | Fe-O def^a,f^ |
| ---- | ---- | 605 | ---- | Fe-O def^a,f^ |
| ---- | ---- | 495 | ---- | Fe-O bond^a,e^ |
| ---- | ---- | 406 | ---- | Fe-O def^a,f^ |
| 391 | ---- | ---- | ---- | Calcite |
| ---- | 344 | ---- | ---- | Cinabbarite^g,h^, HgS^g,h^ |
| ---- | ---- | 289 | ---- | Fe-O def^a,g^ |
| 280 | ---- | ---- | ---- | Calcite^a,f^ |
| ---- | 253 | ---- | ---- | Cinabbarite^g,h^, HgS^g,h^ |

^a^Smith DC, Bouchard M, Lorblanchet M: *J Raman Spectrosc* 1999, 30:347-354.

^b^Alfè M, Gargiulo V, Di Capua R, Chiarella F, Rouzaud JN, Vergara A, Ciajolo A: *ACS Appl Material & Interface* 2012, 4:4491-4498.

^c^Tarantilis PA, Beljebbar A, Manfait M, Polissiou M: *Spectrochim Acta A Mol Biomol Spectrosc* 1998, 54:651-657.

^d^Schulz H, Baranska M, Baranski R: *Biopolimers* 2005, 77:212-221.

^e^Aguayo T, Clavijo E, Eisner F, Ossa-Izquierdo C, Campos-Vallette MM: *J Raman Spectrosc* 2011, 42:2143-2148

^f^Maguregui M, Knuutinen U, Martínez-Arkarazo I, Giakoumaki A, Castro K, Madariaga JM: *J Raman Spectrosc* 2012, 43:1747-1753.

^g^Baraldi P, Baraldi C, Curina R, Tassi L, Zannini P: *Vibrational Spectroscopy* 2007, 43:420-426.

^h^Clark RJH, Gibbs PJ, Seddon KR, Brovenko NM, Petrosyan YA: *J Raman Spectrosc* 1997, 28:91-94.

**Table S2. Frequencies (cm^-1^) of major infrared bands observed in the polar fraction extracted from Pompeii’s samples and peak assignments**

| Frequency cm^-1^ | | | | Predicted peak |
| --- | --- | --- | --- | --- |
| 1^st^ | 2^nd^ | 3^rd^ | 4^th^ |  |
| ---- | 3749 | 3743 | ---- | *ν*OH stretch |
| 3354 | 3374 | 3354 | 3360 | *ν*NH stretch |
| 2929-2885 | 2930-2885 | 2926-2879 | 2933-2891 | *ν_as_*CH_3_ - *ν_as_*CH_2_ -*ν_s_*CH_2_ |
| 2724 | 2717 | 2720 | 2717 | C-H strech. aldehyde |
| ---- | 2512 | ---- | ---- | C=O stretch calcite (carbonate) |
| ---- | 1796 | ---- | ---- | C=O stretch calcite (carbonate) |
| ---- | 1650 | 1653 | 1682 | C=O stretch |
| 1603 | 1593 | 1600 | 1612 | C=O stretch amino acid |
| ---- | 1454 | 1457 | 1422 | Metal fatty-acid complexes |
| 1416 | 1432 | 1346 | 1343 | CH2 wagging vib CH2 twist vib |
| 1359 |  |  |  |  |
| 1245 | 1242 | 1258 | ---- | Stretching vibration of the C-O ester groups  Metal amino-acid complexes |
| 1083 | 1090 | 1090 | 1099 | Si-O stretch. OH def vib  C-O stretch |
| 1039 | 1042 | 1039 | 1054 | OH def. vib. C-O stretch |
| 966 | 969 | 973 | 969 | C-H bend |
| 916 | 916 | 919 | 916 | C-O stretching for disaccharides  and polysaccharides |
| 868 | 874 | 865 | 874 | C=O carbonate bend |
| 795 | 792 | 792 | 792 | N-H def. primary amine |
| 726 | 717 | ---- | 729 | δ(CH2) C=O carbonate bend |

**Table S3. Frequencies of major infrared bands observed in the non-polar fraction extracted from Pompeii’s samples and peak assignments**

| Frequency cm^-1^ | | | | Predicted peak |
| --- | --- | --- | --- | --- |
| 1^st^ | 2^nd^ | 3^rd^ | 4^th^ |  |
| 3287 | ---- | ---- | ---- | The O–H absorption band |
| Region of hydrogen’s stretching | | | |  |
| 2948 | 2952 | 2958 | 2953 | *ν_as_*CH_3_ |
| 2920 | 2920 | 2920 | 2920 | *ν_as_*CH_2_ |
| 2847 | 2850 | 2850 | 2850 | *ν_s_*CH_2_ |
| Region of double bond’s stretching | | | |  |
| 1738 | 1739 | 1736 | 1736 | Ester carbonyl functional group  Free amino acids CH stretch |
| 1644 | ---- | ---- | ---- | C=O stretch  C=C stretching vibration |
| 1600 | 1600 | 1596 | 1603 | Metal amino-acid complexes |
| 1558 | ---- | ---- | ---- | C=O stretch |
| Region of other bonds deformations and bendings | | | |  |
| ---- | 1489-1460 | 1488-1463 | 1495-1448 | Bending vibrations of the CH2 and CH3 aliphatic groups |
| 1448 |  |  | ---- | C-H bending |
| 1356 | 1375 | 1375 | 1378 | Bending vibrations of CH2 groups  Metal fatty-acid complexes |
| ---- | 1242 | 1245 | 1242 | Stretching vibration of the C-O ester groups  Metal amino-acid complexes |
| 1235 | ---- | ---- | ---- | C-H bending |
| Other characteristic peaks | | | |  |
| ---- | 1178 | 1185 | 1185 | CH_2_/NH_2_ twist vib |
| 1083 | ---- | 1080 | ---- | C-O stretch |
| ---- | 1023 | 1026 | 1026 | OH def. vib. C-O stretch |
| 976 | 969 | 969 |  | C-H bend |
| 773 | 754 | 874 |  | CH_2_ rocking vib |
| 694 | 704 | 697 | 697 | Overlapping of the CH_2_ rocking  vibration and the out-of-plane vibration of cis-disubstituted olefins |

**Free amino acids analysis**

**Table S4. Profiles of free amino acids found in the four styles of Pompeian paintings**

| Amino | Ret. Time | 1^st^ style^(a)^ | | 2^nd^ style^(a)^ | | 3^rd^ style^(a)^ | | 4^th^ style^(a)^ | |
| --- | --- | --- | --- | --- | --- | --- | --- | --- | --- |
| acid | min | mg/Kg | % | mg/Kg | % | mg/Kg | % | mg/Kg | % |
| Asp | 1.38 | 0.210 | 5.1 | 0.410 | 5.9 | 0.493 | 2.2 | 0.261 | 2.0 |
| Glu | 1.67 | 0.632 | 15.2 | 0.750 | 10.7 | 2.841 | 12.7 | 1.557 | 11.9 |
| Asn | 3.10 | 0.271**^(b)^** | 6.5 | 0.321**^(b)^** | 4.6 | 0.244**^(b)^** | 1.1 | 0.163**^(b)^** | 1.2 |
| Ser | 3.56 | 0.587 | 14.1 | 0.870 | 12.5 | 0.497 | 2.2 | 0.565 | 4.3 |
| Gln | 4.19 | 0.296 | 7.1 | 0.115 | 1.6 | 0.350 | 1.6 | 0.244 | 1.9 |
| Gly | 5.13 | 0.646 | 15.6 | 0.702 | 10.1 | 4.919 | 22.1 | 1.455 | 11.2 |
| Thr | 5.37 | 0.138 | 3.3 | 0.375 | 5.4 | 0.179 | 0.8 | 0.150 | 1.2 |
| Cit | 5.78 | 0.059**^(b)^** | 1.4 | 0.326 | 4.7 | 0.145**^(b)^** | 0.7 | 0.105**^(b)^** | 0.8 |
| Arg | 6.81 | 0.111 | 2.7 | 0.052**^(b)^** | 0.7 | 0.102 | 0.5 | 0.065 | 0.5 |
| Ala | 7.36 | 0.350 | 8.4 | 0.735 | 10.5 | 4.356 | 19.5 | 2.616 | 20.1 |
| Tyr | 9.67 | 0.149**^(b)^** | 3.6 | 0.311 | 4.5 | 0.370 | 1.7 | 0.298 | 2.3 |
| Val | 12.94 | 0.283 | 6.8 | 0.469 | 6.7 | 1.205 | 5.4 | 1.133 | 8.7 |
| Met | 13.33 | n.d. | 0 | 0.071**^(b)^** | 1.0 | 0.071**^(b)^** | 0.3 | 0.055**^(b)^** | 0.4 |
| Phe | 15.10 | 0.133**^(b)^** | 3.2 | 0.312 | 4.5 | 0.911 | 4.1 | 0.517 | 4.0 |
| Ile | 15.27 | 0.112**^(b)^** | 2.7 | 0.282 | 4.0 | 0.732 | 3.3 | 0.748 | 5.7 |
| Orn | 15.71 | n.d. | 0 | n.d. | 0 | 0.106**^(b)^** | 0.5 | 0.104 | 0.8 |
| Leu | 16.09 | 0.135**^(b)^** | 3.2 | 0.477 | 6.8 | 1.464 | 6.6 | 1.229 | 9.4 |
| Lys | 16.61 | n.d. | 0 | n.d. | 0 | 0.083**^(b)^** | 0.4 | n.d. | 0 |
| 5-OH-Pro | 17.59 | 0.135**^(b)^** | 3.3 | 0.077**^(b)^** | 1.1 | 0.332**^(b)^** | 1.5 | 0.088**^(b)^** | 0.7 |
| Pro | 20.85 | 0.205 | 4.9 | 0.438 | 6.3 | 3.258 | 14.6 | 1.936 | 14.8 |

n.d.: not detected; (a): mean values of two determinations; (b): value < LOD.

**Table S5.** Amino acid composition from 15 representative sample proteins

|  | Arg | Lys | Glu | Asp | Tyr | Phe | Met | Val | Pro | Thr | Ala | Ser | Gly | Ile/Leu |
| --- | --- | --- | --- | --- | --- | --- | --- | --- | --- | --- | --- | --- | --- | --- |
| Milk | 3.45 | 7.55 | 19.94 | 7.22 | 4.60 | 4.60 | 2.39 | 6.37 | 9.22 | 4.30 | 3.28 | 5.18 | 2.01 | 5.2 |
| Lysozim | 11.53 | 5.18 | 3.92 | 16.53 | 3.25 | 2.83 | 1.87 | 4.36 | 1.27 | 4.99 | 5.27 | 6.08 | 5.18 | 8.99 |
| Conalbumin | 6.68 | 8.80 | 10.47 | 11.70 | 4.05 | 5.01 | 1.79 | 7.21 | 4.31 | 5.19 | 3.87 | 5.54 | 5.01 | 7.92 |
| Ovomucoid | 4.84 | 7.85 | 8.50 | 17.01 | 4.16 | 3.81 | 1.24 | 7.85 | 3.56 | 7.20 | 3.01 | 5.49 | 4.97 | 13.46 |
| Avidin | 8.14 | 7.76 | 8.27 | 12.15 | 1.10 | 7.39 | 1.77 | 5.26 | 2.05 | 13.20 | 0.00 | 5.64 | 5.76 | 15.09 |
| Fosfvitin | 7.27 | 8.94 | 5.15 | 6.67 | 0.15 | 0.91 | 0.45 | 1.67 | 1.52 | 2.10 | 2.27 | 50.00 | 2.42 | 10.99 |
| Vitellin | 8.59 | 7.06 | 11.25 | 8.28 | 3.89 | 4.09 | 2.86 | 6.34 | 4.50 | 4.80 | 4.09 | 11.45 | 2.86 | 12.14 |
| Cocrodil | 18.20 | 4.64 | 6.65 | 4.15 | 0.30 | 1.62 | 0.59 | 1.41 | 11.73 | 2.00 | 10.41 | 3.85 | 30.26 | 8.54 |
| Piton | 18.44 | 5.11 | 5.77 | 4.44 | 0.16 | 1.32 | 0.56 | 1.87 | 11.05 | 1.66 | 11.58 | 4.03 | 29.25 | 13.03 |
| Toad | 17.72 | 5.26 | 7.02 | 4.96 | 0.55 | 1.74 | 1.01 | 1.97 | 9.89 | 2.40 | 8.86 | 5.97 | 27.05 | 2.28 |
| Chiken | 17.02 | 3.60 | 7.03 | 4.56 | 0.32 | 1.36 | 0.58 | 1.88 | 12.29 | 1.80 | 10.90 | 2.71 | 31.37 | 14.21 |
| Gluten | 3.50 | 1.65 | 34.49 | 3.81 | 3.29 | 5.15 | 1.75 | 4.32 | 12.49 | 2.57 | 2.27 | 4.74 | 3.50 | 2.88 |
| Gliadin | 2.15 | 0.43 | 42.35 | 2.63 | 2.58 | 6.07 | 1.10 | 3.54 | 14.53 | 1.72 | 2.01 | 4.30 | 1.39 | 3.45 |
| Glutenin | 3.01 | 0.87 | 41.68 | 2.40 | 3.54 | 4.29 | 1.39 | 3.33 | 12.09 | 2.47 | 2.46 | 5.74 | 2.81 | 3.85 |
| Albumin | 9.11 | 4.96 | 19.85 | 7.74 | 5.07 | 5.39 | 0.00 | 5.30 | 9.56 | 2.87 | 4.51 | 5.37 | 5.13 | 3.29 |

**
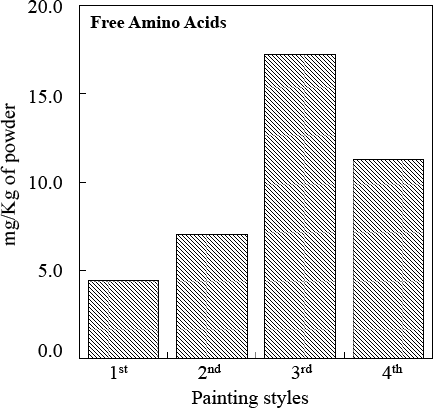
**

**Figure S1. Total amount of free amino acid in Pompeian wall paintings.**

**Mass spectrometry analysis of sugars and fatty acids.**

**Table S6. Carbohydrates composition of complex sugars extracted from Pompeian wall painting samples**

|  | **1^st^ style** | **2^nd^ style** | **3^rd^ style** | **4^th^ style** |
| --- | --- | --- | --- | --- |
|  | mg/kg | | | |
| Arabinose | 21.98 | 4.35 | 10.44 | 7.65 |
| Fucose | 50.19 | 14.05 | 42.05 | 6.92 |
| Xylose | 316.00 | 81.16 | 220.66 | 39.21 |
| Galactose | 21.96 | 5.42 | 4.90 | 2.06 |
| Glucose | 60.72 | 12.57 | 59.00 | 6.51 |
| Galacturonic acid | 14.92 | 2.78 | 1.40 | 1.43 |
| Myo-inositol | 24.01 | 1.14 | 0.95 | n.d |
|  | % | | | |
| Arabinose | 4.31 | 3.58 | 3.08 | 11.99 |
| Fucose | 9.84 | 11.57 | 12.39 | 10.84 |
| Xylose | 61.99 | 66.82 | 65.01 | 61.48 |
| Galactose | 4.31 | 4.46 | 1.44 | 3.23 |
| Glucose | 11.91 | 10.35 | 17.38 | 10.21 |
| Galacturonic acid | 2.93 | 2.28 | 0.41 | 2.24 |
| Myo-inositol | 4.71 | 0.94 | 0.28^(a)^ | 0.06^(b)^ |
| Ratios of carbohydrate composition* | | | | |
| Fucose/Arabinose + Xylose | 0.15 | 0.16 | 0.18 | 0.15 |
| Galactose/Arabinose + Xylose | 0.07 | 0.06 | 0.02 | 0.04 |

* Riedo C, Scalarone D, Chiantore O, *Anal Bioanal Chem* 2010, **396**:1559-1569.

^(a)^ LOQ = 0.69; ^(b)^ LOD = 0.37

| **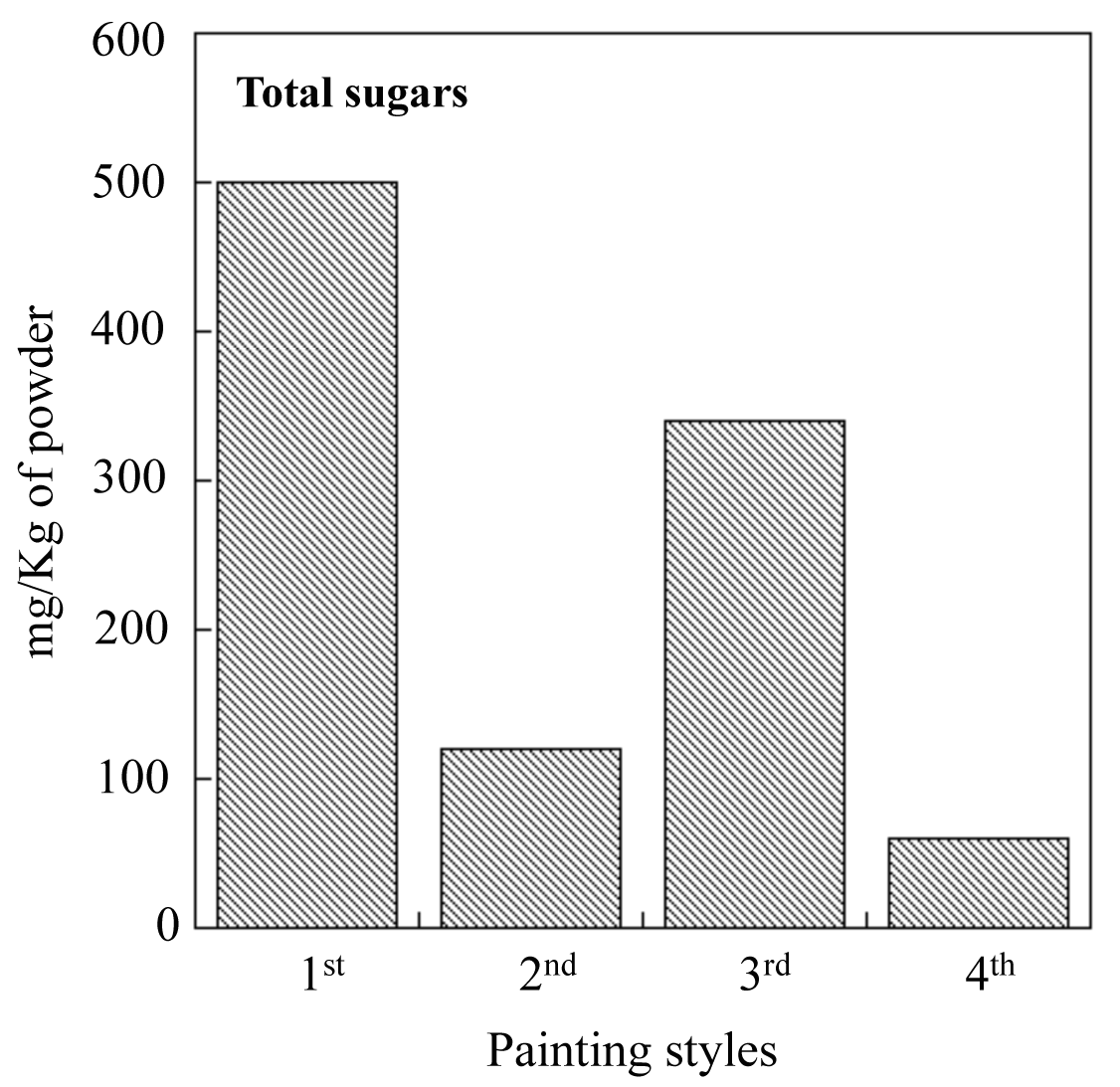** | 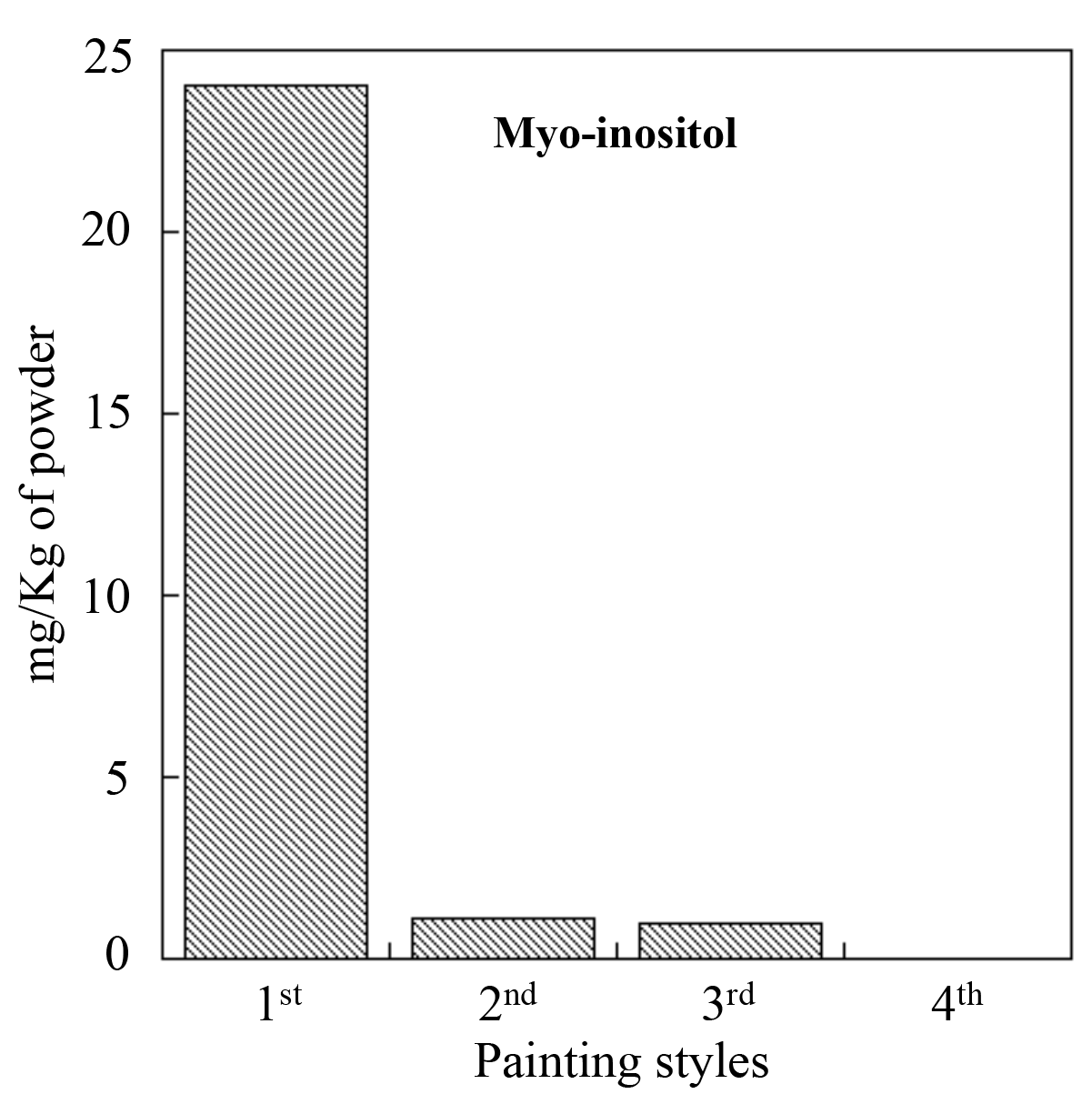 |
| --- | --- |
| **Figure S2. Total amount of sugars in Pompeian wall paintings.** | **Figure S3. Total amount myo-inositol in Pompeian wall paintings.** |

**Table S7.** Fatty acids composition of lipids extracted from Pompeian wall painting samples

|  | 1^st^ style | 2^nd^ style | 3^rd^ style | 4^th^ style |
| --- | --- | --- | --- | --- |
|  | mg/Kg powder | | | |
| C16:1 | 2.6 | 1.3 | 0.82^(a)^ | 1.7 |
| C16:0 | 63.4 | 44.8 | 39.1 | 30.2 |
| C18:2 | 10.9 | 2.7 | 5.8 | 3.2 |
| C18:1 | 15.5 | 5.3 | 6.5 | 6.4 |
| C18:0 | 69.8 | 40.2 | 46.7 | 31.3 |
|  | % | | | |
| C16:1 | 1.6 | 1.4 | 0.8 | 2.4 |
| C16:0 | 39.1 | 47.5 | 39.5 | 41.5 |
| C18:2 | 6.7 | 2.9 | 5.8 | 4.3 |
| C18:1 | 9.6 | 5.6 | 6.6 | 8.9 |
| C18:0 | 43.0 | 42.6 | 47.2 | 43.0 |

^(a)^ LOQ = 0.79; LOD = 0.47

**
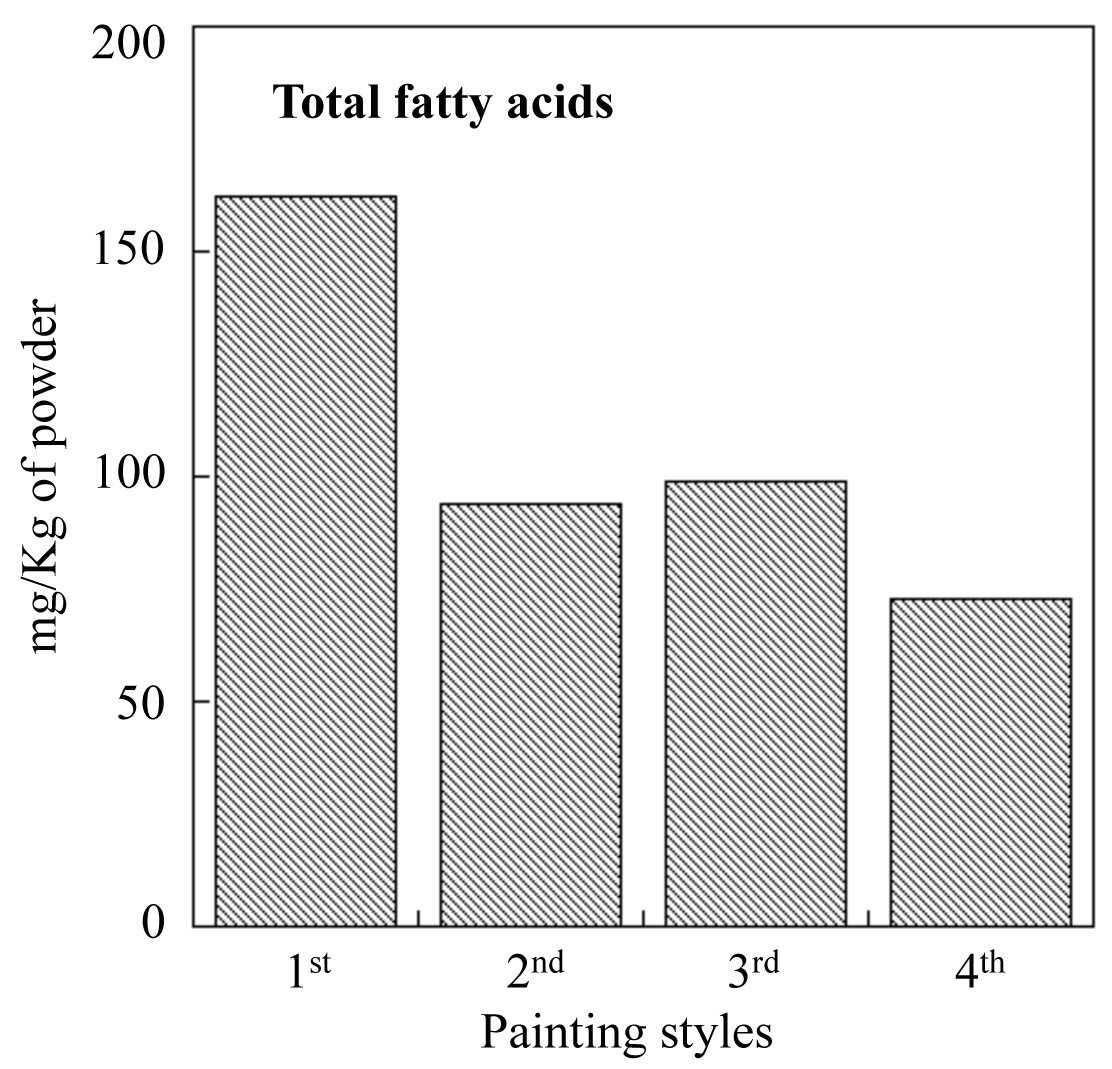
**

**Figure S4.** **Total fatty acids in Pompeian wall paintings.** The fatty acids were determined after hydrolysis of non-polar fractions.

**LOD and LOQ values for the analyzed compounds.**

| **Table S8. LOD and LOQ values of Amino acids, sugars and lipids** | | | | | |  |
| --- | --- | --- | --- | --- | --- | --- |
| Amino acids | LOD | LOQ |  | Sugars | LOD | LOQ |
|  | mg/Kg | mg/Kg |  |  | mg/Kg | mg/Kg |
| Arg | 0,061 | 0,078 |  | Arabinose | 0,520 | 0,706 |
| Cit | 0,198 | 0,280 |  | Fucose | 0,428 | 0,587 |
| Lys | 0,247 | 0,347 |  | Xylose | 0,362 | 0,475 |
| Orn | 0,777 | 1,197 |  | Galactose | 0,386 | 0,523 |
| Asn | 0,543 | 0,824 |  | Glucose | 0,423 | 0,577 |
| Gln | 0,060 | 0,073 |  | Galacturonic acid | 0,445 | 0,618 |
| Glu | 0,106 | 0,203 |  | Myoinositol | 0,369 | 0,533 |
| Asp | 0,023 | 0,031 |  |  |  |  |
| Tyr | 0,194 | 0,293 |  |  |  |  |
| Phe | 0,137 | 0,203 |  |  |  |  |
| Met | 0,107 | 0,156 |  |  |  |  |
| Ile | 0,134 | 0,202 |  |  |  |  |
| Leu | 0,174 | 0,267 |  |  |  |  |
| Val | 0,073 | 0,105 |  | Lipids | LOD | LOQ |
| Pro | 0,014 | 0,024 |  |  | mg/Kg | mg/Kg |
| Thr | 0,069 | 0,092 |  | C16:1 | 0,445 | 0,567 |
| 5-OH-Pro | 1,054 | 1,713 |  | C16:0 | 0,492 | 0,617 |
| Ala | 0,060 | 0,088 |  | C18:2 | 0,452 | 0,604 |
| Ser | 0,051 | 0,061 |  | C18:1 | 0,301 | 0,375 |
| Gly | 0,032 | 0,044 |  | C18:0 | 0,422 | 0,576 |
